# Respiratory support for patients with COVID‐19: A national survey of respiratory departments in England

**DOI:** 10.1111/crj.13535

**Published:** 2022-09-02

**Authors:** Sam Bartlett‐Pestell, Annakan V. Navaratnam, Ini Adelaja, Martin Allen

**Affiliations:** ^1^ NHS England and Improvement; Respiratory Registrar St George's University Hospitals NHS Trust London UK; ^2^ Getting it Right First Time, NHS England and Improvement; ENT Registrar University College Hospital London London UK; ^3^ NHS England and Improvement, Kingston Hospital NHS Foundation Trust London UK; ^4^ Getting it Right First Time, NHS England and Improvement; Consultant Respiratory Physician University Hospital North Midlands NHS Trust Stoke UK

**Keywords:** COVID‐19, non‐invasive ventilation, respiratory support, surge planning

## Abstract

**Objective:**

We developed a national survey to assess the changes implemented by respiratory departments across England in response to the first wave of the COVID‐19 pandemic.

**Methods:**

An online survey was sent to the respiratory clinical leads in 132 NHS trusts in England. The survey was open between 10 August 2020 and 25 September 2020.

**Results:**

Fifty‐three responses (42%) are included in our results.

The total number of non‐critical care led Level 2 beds (requiring care for single organ failure—capable of managing continuous positive airways pressure, CPAP) increased by 159% at peak COVID activity from levels prior to COVID‐19. CPAP was used solely in side‐rooms in 9% of sites, and 57% and 31% of sites used CPAP in closed bays and closed wards, respectively.

Fifteen sites (28%) reported shortages of non‐vented non‐invasive ventilation (NIV) masks and 12 sites (23%) CPAP machines. There was regional variation.

**Conclusions:**

The number of beds capable of managing patients requiring CPAP increased significantly. We found deviations from previous standards of care, which likely reflects the pressure faced by hospitals in managing patients with COVID‐19. The regional variation in equipment shortages suggests moving resources between regions may have been beneficial.

## Graphical abstract

We developed a national survey to assess the changes implemented by respiratory departments across England in response to the first wave of the COVID‐19 pandemic. The number of beds capable of managing patients requiring CPAP increased significantly. We found deviations from previous standards of care which likely reflects the pressure faced by hospitals in managing patients with COVID‐19.

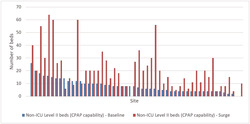

## 1 INTRODUCTION

The COVID‐19 pandemic has demanded rapid change and adaptation from healthcare teams and organisations.

Best practice for managing patients with COVID‐19 has rapidly evolved. From March 2020 there was an increase in clinical and operational guidance for managing patients during the pandemic, and in the United Kingdom both NHS England and the British Thoracic Society (BTS) published guidance on the provision of ventilatory support (These have subsequently been withdrawn and updated guidance is available on the BTS and National Institute for Health and Care excellence (NICE) websites.).^1^, ^2^

We developed a national survey to assess the changes to practice implemented by respiratory departments across England in response to the COVID‐19 pandemic and help better prepare for future surges in hospital admissions due to COVID‐19 and other viral respiratory illnesses.

## 2 METHODS

An online survey was created using Citizen Space (Delib, Bristol, UK) by a team including clinicians (SBP, AVN, IA, MA), a data analyst (MC) and a project manager with extensive experience in survey design (PSS). The survey was tested by clinicians with previous experience of clinical survey implementation (SBP, AVN, IA) and a respiratory consultant (MA). A link to the survey was sent to the respiratory clinical leads in 132 NHS trusts in England that provide respiratory care, compiled by Getting it Right First Time (GIRFT). The survey was open between 10 August 2020 and 25 September 2020.

Data analysis was conducted in Microsoft Excel. Quantitative data were reported with percentages, ranges, medians and inter‐quartile ranges, as appropriate. Fishers Exact Test was used to calculate the *p* values for regional differences in equipment shortages. Data were omitted if deemed erroneous independently by three clinicians (SBP, AK, IA).

There was no patient or public involvement in this survey. The study was deemed to be an evaluation of service and therefore ethical approval was not sought.

## 3 RESULTS

Fifty‐eight (43.9%) responses were received, and five sites opted‐out of having their data included in peer‐review publications. We present the results of 53 responses.

### 3.1 Bed capacity

**FIGURE 1:**
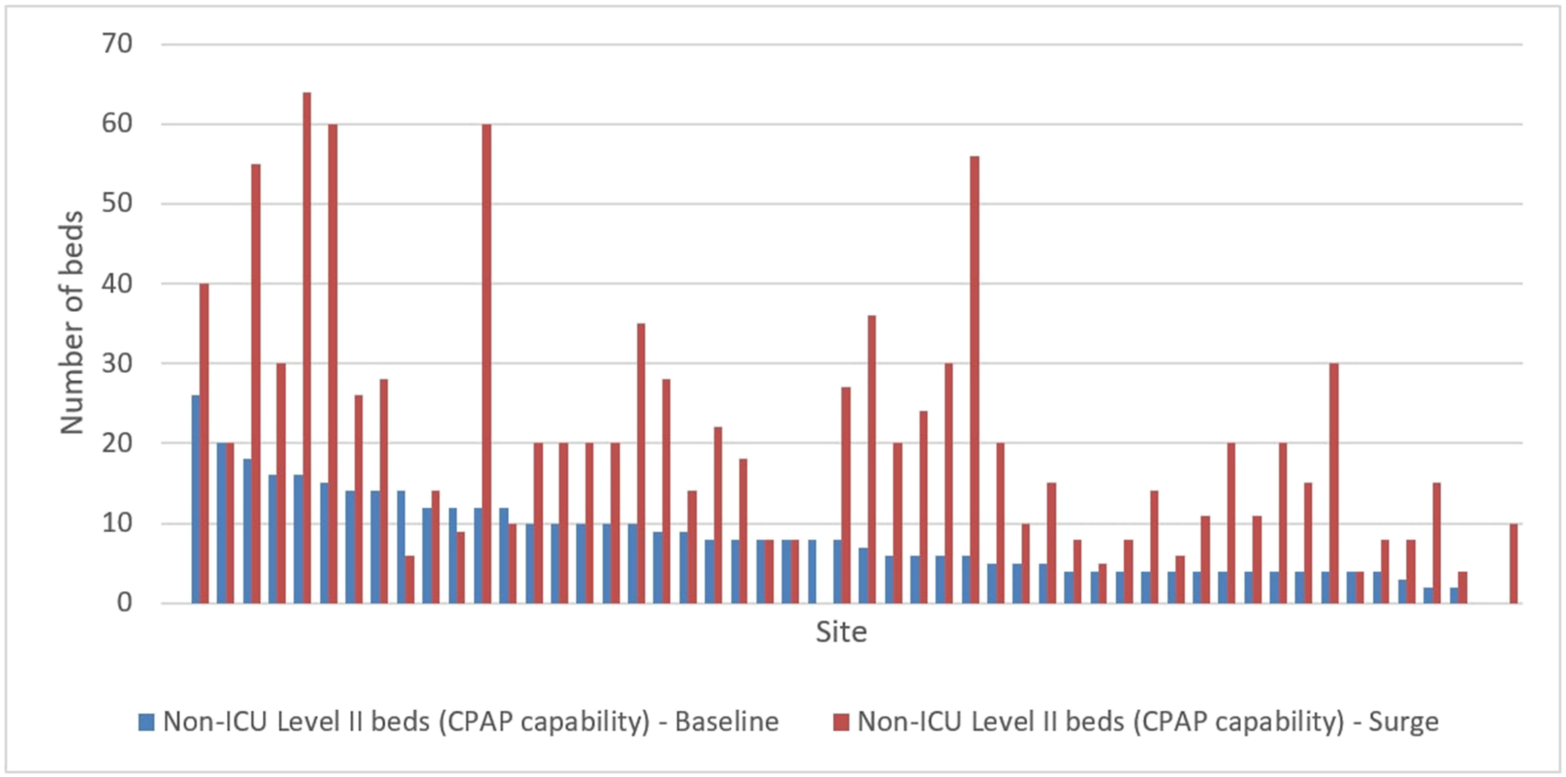
Level 2 bed capacity at baseline and surge

There were a reported 422 non‐critical care led level 2 beds (beds capable of managing patients requiring single organ support such as continuous positive pressure airway pressure, CPAP) prior to COVID‐19 (data from 52 sites) (median 7.5; range 0–26; IQR 4–10.5), which increased to 1070 at the peak of COVID‐19 activity (the day with highest number of COVID‐19 admissions) (data from 51 sites) (median 20; range 0–64; IQR 9.5–27.5); an increase of 158.5%. 3/51 sites reported fewer non‐critical led level 2 beds at the peak of COVID‐19 activity. Figure 1 shows the variation in non‐ICU level 2 beds across sites.

### 3.2 Respiratory support

**FIGURE 2:**
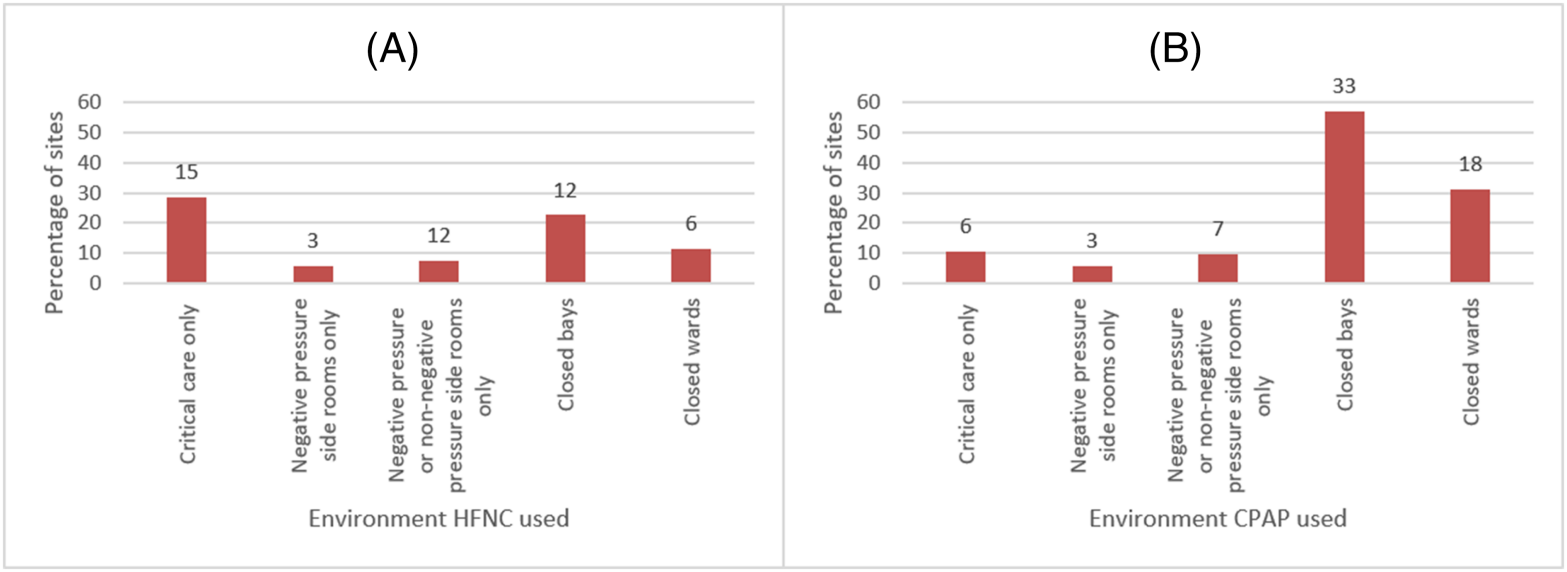
Environments high flow nasal cannula (HFNC) and continuous positive airways pressure (CPAP) used. (A) Environments HFNC used for patients with COVID‐19, *n* = 53. (B) Environments CPAP used for patients with COVID‐19, *n* = 53. *Numbers above the bars indicate number of sites.*

The 52/53 (91.1%) sites used CPAP for patients with COVID‐19. The 32/53 (60.4%) sites used high flow nasal cannula (HFNC) for patients with COVID‐19. Figure 2 shows the environments in which CPAP and HFNC were used for patients with COVID‐19 (Siderooms refer to single occupancy rooms with or without an antechamber; closed bays refer to multiple occupancy rooms [usually 4–8 patients] where patients would be cohorted and closed wards consist of multiple bays and potentially some siderooms where patients would be cohorted.). HFNC was limited more to critical care when compared with CPAP and CPAP was used more frequently on closed bays and wards.

The 24/53 sites (45.3%) used CPAP for patients with COVID‐19, who in other circumstances may have been intubated. Proning was used for at least some hypoxic patients before trialling CPAP in 48/53 (90.6%) sites. In 5/53 sites (9.4%) this was routine for all hypoxic patients.

**FIGURE 3:**
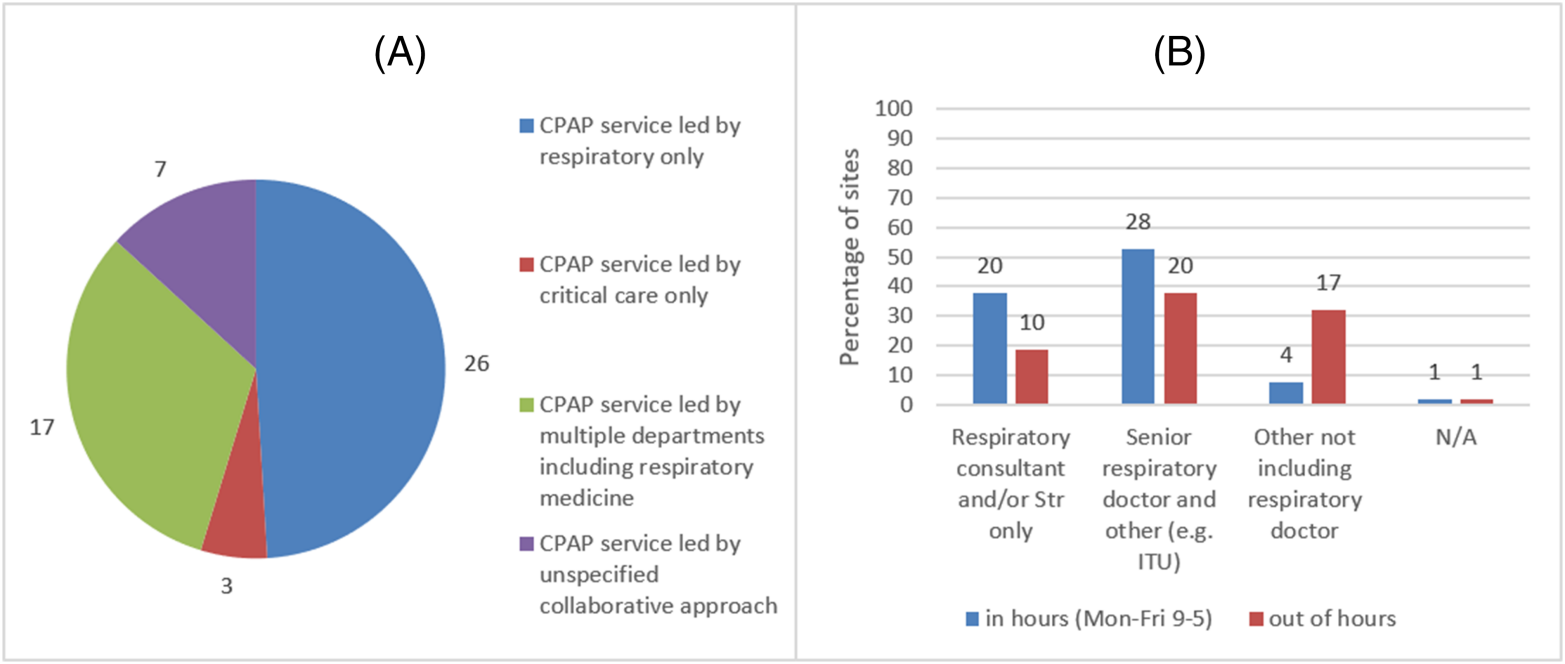
Continuous positive airways pressure (CPAP) leadership. (A) Responsibility for leading CPAP service for Patients with COVID‐19, *n* = 53. (B) Key decision maker for initiating CPAP in and out‐of‐hours, *n* = 53. Str = specialty registrar; senior respiratory doctor = consultant or Str. *Numbers above the bars and next to the pie chart indicate number of sites.*

Figure 3 shows the leadership for the CPAP service and decision making for CPAP both in and out‐of‐hours. The 26/53 (49.1%) sites reported the CPAP service for patients with COVID‐19 was solely led by respiratory teams. A further 17/53 (32.1%) sites reported respiratory teams led the service in collaboration with other teams.

### 3.3 Equipment

Table 1 shows the regional differences in equipment shortages. London had more sites with equipment shortages (with statistical significance) than the Midlands, North East and Yorkshire and the South West. 15/53 sites (28.3%) reported shortages of non‐vented NIV masks (we did not collect data on the type of mask, e.g., full face and hood) and 12/53 sites (22.6%) CPAP machines. 20/53 (37.7%) sites used low flow domiciliary CPAP machines for inpatients with COVID‐19 requiring respiratory support.

**TABLE 1:** Equipment shortages

Geographical region	No equipment shortage	Shortage of at least one item^a^	Total	*p* value(compared with London)
EOE	1	1	2	0.264
London	1	11	12	
Midlands	5	3	8	0.017
NEY	5	4	9	0.028
NW	3	3	6	0.078
SE	6	3	9	0.429
SW	5	2	7	0.009
Total	26	27	53	

Abbreviations: NEY, North East and Yorkshire; NW, North West; SE, South East; SW, South West.

^a^ Vented non‐invasive ventilation (NIV) masks, non‐vented NIV masks, continuous positive airways pressure (CPAP) machines, venturi oxygen masks and non‐rebreathe oxygen masks.

## 4 DISCUSSION

Our national survey of respiratory departments showed that there had been significant changes made due to the COVID‐19 pandemic. Not surprisingly, given the scale of the pandemic, the number of non‐critical care level 2 beds dramatically increased. It is probable that respiratory teams were instrumental in increasing, and managing patients occupying, non‐critical care level 2 beds as evidenced by the number of sites that reported respiratory consultants led COVID‐19 CPAP services.

Due to the high numbers of patients being treated during the first wave, and the rapid change in capacity and workforce, some traditional practices of care delivery changed. Under 10% of units delivered CPAP for patients with COVID‐19 in side‐rooms only and over half delivered it in closed bays, where previously for infections such as influenza managing patients in side‐rooms was standard practice.^3^ This highlights the pressure on sites for clinical space to deliver CPAP.

The evidence surrounding CPAP changed during the first wave of COVID‐19 with reports from Italy emerging that it was beneficial.^4^ Twenty‐four units (45.3%) reported using CPAP for patients who in other circumstances would have been intubated. This might reflect the evolving knowledge base during the first wave of the pandemic where CPAP was used more frequently for some patients who initially would have been intubated,^5^ or it may represent the pressure hospitals faced in managing patients with COVID‐19.

The proning of patients previously has been reserved for intubated patients. The use of proning showed some benefits on gas exchange in patients treated with CPAP with hoods,^6^ and improved oxygen saturations in patients on oxygen.^7^ In our survey almost all units (90.6%) used proning for at least some hypoxic patients prior to initiating CPAP and 5 units used this strategy for all patients.

We found that over 20% of sites reported a shortage of CPAP machines and almost a third reported shortages of non‐vented masks during the first wave. There was some regional variation, which suggests moving resources between regions may have been beneficial.

### 4.1 Limitations

The survey used self‐reported data, which in some cases might be inaccurate. This survey asked respondents to report non‐critical care led level 2 bed data, and therefore, critical care level 2 bed data are not included. This may have led to underestimation of a hospital trust's total level 2 capacity.

## 5 CONCLUSIONS

We present a large national survey of respiratory departments during the first wave of the COVID‐19 pandemic. We found that the number of beds capable of managing patients requiring CPAP increased significantly and in half of the sites the CPAP service was led solely by respiratory consultants. We found deviations from previous standards of care, which may reflect the pressure faced by the hospitals in managing patients with COVID‐19. Equipment shortages were common in some areas and moving resources between regions may have been beneficial.

## CONFLICT OF INTEREST

The authors have no competing or financial interests to declare.

## ETHICS STATEMENT

The study was deemed to be an evaluation of service, and therefore, ethical approval was not sought.

## AUTHOR CONTRIBUTIONS

Dr Sam Bartlett‐Pestell, Mr Annakan Navaratnam and Dr Ini Adelaja made substantial contributions to the design of the work, interpretation of data, drafted the work and have final approval for publication. Dr Martin Allen made substantial contributions to the design of the work, interpretation of data, revisions to the work and has final approval for publication.

## Data Availability

The data that support the findings of this study are available on request from the corresponding author. The data are not publicly available due to privacy or ethical restrictions.
